# Comparative Assessment of Post-Fatigue Resistance of Mandibular First Molars Restored With Polyether Ether Ketone and Lithium Disilicate Endocrowns

**DOI:** 10.1155/tswj/8648372

**Published:** 2024-11-27

**Authors:** Mehrak Amjadi, Mohammad Raouf Safari, Ramin Dini Torkamani, Mohammadsepehr Kheiri Manjili, Davoud Jamshidi, Fatemeh Sobhan, Soolmaz Heidari

**Affiliations:** ^1^Department of Prosthodontics, School of Dentistry, Qazvin University of Medical Sciences, Qazvin, Iran; ^2^Dental Research Center, School of Dentistry, Golestan University of Medical Sciences, Gorgan, Iran; ^3^Student Research Committee, School of Dentistry, Qazvin University of Medical Sciences, Qazvin, Iran; ^4^Department of Endodontics, School of Dentistry, Qazvin University of Medical Sciences, Qazvin, Iran; ^5^Department of Materials Engineering, Faculty of Technical and Engineering, Imam Khomeini International University, Qazvin, Iran; ^6^Department of Operative Dentistry, School of Dentistry, Qazvin University of Medical Sciences, Qazvin, Iran

**Keywords:** endocrown, fracture resistance, lithium disilicate, polyether ether ketone

## Abstract

**Background and Objectives:** Contemporary dentistry focuses on more conservative treatment options such as endocrown restorations and application of dental materials with higher resemblance to tooth structure. Polyether ether ketone (PEEK) polymer is a material used for the fabrication of endocrowns. This study aimed to compare the post-fatigue resistance (PFR) of mandibular first molars restored with PEEK and lithium disilicate (LS_2_) endocrown restorations.

**Materials and Methods:** This in vitro, experimental study was conducted on 20 human mandibular first molars with similar dimensions. The teeth were prepared for endocrown restoration and were assigned to two groups (*n* = 10) of PEEK and LS_2_ endocrowns. After fabrication by the computer-aided design and computer-aided manufacturing (CAD-CAM) technique, the restorations were cemented with resin cement. Next, the teeth underwent 15,000 thermal cycles followed by cyclic loading with 600,000 cycles of compressive force (100 N, 4 Hz) and were then subjected to compressive load application in a universal testing machine. The load causing endocrown failure was recorded as the PFR of the respective restoration. The failure mode was also inspected under a light microscope. Data were analyzed by the independent *t*-test and also chi-square, Mann–Whitney, and Fisher's exact tests (*α* < 0.05).

**Results:** The teeth with PEEK endocrowns showed significantly higher PFR than those with LS_2_ endocrowns. Irreparable fractures were dominant in both groups.

**Conclusion:** PEEK may serve as a suitable alternative to LS_2_ for the fabrication of endocrown restorations.

## 1. Introduction

Reconstruction of endodontically treated teeth has been the topic of numerous investigations; nonetheless, it is still a challenging procedure in clinical dental practice. Factors such as changed composition and extensive loss of tooth structure, which can decrease the quality of adhesion and fracture resistance of teeth, may eventually lead to restoration failure in endodontically treated teeth [1].

The cast post-core crown is the most commonly used restorative technique for reconstructing endodontically treated teeth with little remaining tooth structure [2]. However, despite the reportedly optimal clinical success of this procedure, it cannot be used for certain cases, such as roots with severe curvature, calcified roots, and short clinical crowns, where crown lengthening surgery compromises the prognosis of the tooth [3]. One suggested strategy for such cases is to use all-ceramic overlays or endocrowns as a more conservative approach [4, 5]. Endocrown restorations have advantages such as requiring minimal removal of sound tooth structure, requiring fewer procedural steps and shorter clinical time for fabrication, and higher patient comfort, compared with other treatment options [3, 6, 7]. Endocrown restorations are composed of a coronal part and a cavity part, which is located within the pulp chamber. The cavity part of restoration uses the pulp chamber surfaces to achieve macro and micro mechanical retention through adhesion, which is different from the adhesion mechanism of post and core restorations [8].

Leucite-reinforced feldspathic ceramics, lithium disilicate (LS_2_)–reinforced ceramics, and hybrid ceramics can all be used for the fabrication of endocrown restorations [7, 9]. It appears that LS_2_-reinforced ceramics are among the best materials for the fabrication of endocrowns by the pressing technique or machined technique using the computer-aided design and computer-aided manufacturing (CAD-CAM) technology. Optimal mechanical properties, strong bonding to tooth structure, and excellent esthetics are among the advantages of LS_2_ ceramics [10].

Recently, polyether ether ketone (PEEK) was introduced to dentistry as a high-performance polymer. This polymer belongs to the polyaryletherketone (PAEK) family and has optimal chemical, thermal, and mechanical properties in addition to favorable biocompatibility [11].

It has low solubility in water and optimal dimensional stability [12, 13]. Also, it can be used for restoration fabrication in patients allergic to metals [14]. Considering the optimal mechanical properties of PEEK which can be further improved by addition of different materials, it may be used for the fabrication of dental restorations [15, 16].

This study aimed to compare the cyclic fatigue resistance of endocrowns fabricated from LS_2_ ceramic and PEEK polymer for mandibular first molars.

## 2. Materials and Methods

The composition of the materials used in this study is specified in Table 1.

### 2.1. Tooth Preparation

The intact third molars were selected. The teeth were evaluated with a stereomicroscope to rule out the teeth with any cracks or anomalies. The buccolingual (from the crest of the curvature on the buccal surface to the crest of the curvature on the lingual surface), mesiodistal (from mesial contact area to distal contact area), and occlusocervical (from the highest point of the buccal cusp to cementoenamel junction (CEJ)) dimensions of all teeth were measured with a digital caliper to select teeth with similar dimensions. The teeth with 20% deviation from the mean values were excluded [17, 18].

The teeth were immersed in 0.5% chloramine T solution for disinfection for 1 week. They were then stored in distilled water at 4°C until the experiment. The teeth were mounted in auto-polymerizing acrylic resin to 3 mm below their CEJ along their longitudinal axis by using a surveyor [19]. This was done to simulate the position of natural teeth in bone. Acrylic ring molds with 1.5 cm height were used for this purpose. For maximum standardization of the teeth, they were measured in mesiodistal and buccolingual dimensions by using a digital caliper, and those with similar dimensions were selected for the study.

The selected teeth were randomly divided into two groups. The teeth in Group 1 received endocrown restorations fabricated from PEEK (breCAM.BioHPP, Bredent GmbH & co. KG., Senden, Germany) while the teeth in Group 2 received endocrown restorations fabricated from LS_2_ ceramic (IPS e.max CAD, Ivoclar Vivadent, Schaan, Liechtenstein).

For preparation of teeth, an access cavity was prepared by using a diamond fissure bur. Root canal therapy was performed by using hand files with the step-back technique, and the root canals were obturated with gutta-percha and AH26 sealer (Dentsply, DeTrey, Konstanz, Germany) by the lateral compaction technique. To ensure a hermetic coronal seal, the canal orifice was restored with light-cure restorative glass ionomer (GC Fuji II LC, GC Corporation, Tokyo, Japan). For occlusal reduction, guiding grooves were created in the occlusal surface with 2 mm depth by using a fissure bur. A wheel-shaped bur was then used parallel to the longitudinal axis of the tooth for occlusal clearance such that a flat surface was obtained. Pulp chamber preparation only included elimination of undercuts and creation of 8–10-degree internal taper relative to the longitudinal axis of the tooth. Internal taper was created by using a round-end tapered diamond bur perpendicular to the pulp chamber floor. All internal line angles were rounded, and the pulp chamber depth was measured by a periodontal probe and standardized at 4 mm in all teeth. The pulp chamber depth was measured from the butt joint margin to the bottom of the pulp chamber. For preparation and decortication of the teeth, a standard guideline was meticulously followed to achieve an approximately similar condition for each tooth. The margins were formed as butt-joint with 90-degree angle and no ferrule. All steps were performed by a single operator.

### 2.2. Endocrown Fabrication

All teeth were scanned by a laboratory scanner (inEos X5; Dentsply Sirona, NY, USA) and digital data were collected. Restorations were designed by using the respective software (inLab SW 22 CAD, Dentsply Sirona, NY, USA). All restorations were designed similarly with 2 mm occlusal thickness. The cement thickness was 60 *μ*m in all restorations. The 3D data of restorations were sent to a milling machine (inLab MC X5: Dental Milling Machine, NY, USA) and milling was performed by using LS_2_ and PEEK CAD-CAM blocks. The LS_2_ blocks were heated in a furnace (Programat CS, Ivoclar Vivadent) after milling as instructed by the manufacturer (Table 2).

### 2.3. Cementation Process

After fabrication, all restorations were cemented with resin cement (Multilink N, Ivoclar Vivadent, Liechtenstein) as instructed by the manufacturer. The cementation process was performed in the following two forms for all restorations.

**Figure 1:**
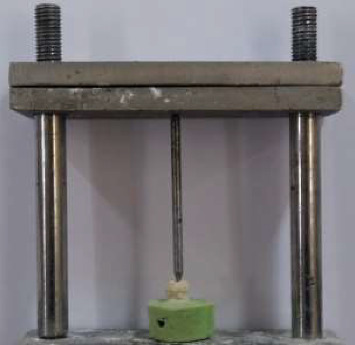
Custom-made device used for cementation.

For cementation of LS_2_ restorations, their intaglio surface was first etched with 5% hydrofluoric acid gel (IPS Ceramic Etching Gel; Ivoclar Vivadent, Liechtenstein) for 20 s, rinsed with water, and then air-dried. Next, one thin layer of silane (Monobond N; Ivoclar Vivadent, Liechtenstein) was applied on the internal restoration surface by a micro-brush and left in air for 60 s and then vigorously dispersed with an air spray. The A and B primers of Multilink N were mixed in 1:1 ratio and applied on the treated surface. To prepare the tooth surface, the teeth were then cleaned with an oil-free and fluoride-free prophylaxis paste, which was followed by rinsing and drying the teeth. The enamel edges were etched with 37% phosphoric acid (N-Etch; Ivoclar Vivadent, Liechtenstein) for 30 s and then rinsed and air-dried. The A and B primers of Multilink N were again mixed in 1:1 ratio, rubbed on the tooth surface for 30 s, and dried with gentle air spray. Resin cement was applied on the restoration surface and inside the teeth. A custom-made device with a simple design was used to ensure equal application of 1 kg force for cementation of restorations (Figure 1). Curing was initially performed for 2 s, and excess cement was removed. Finally, each restoration was light-cured for 20 s from each side with a LED curing unit (LED B, woodpecker, China) with 430–480 nm wavelength and 1200 mW/cm^2^ light intensity. Liquid strip (Ivoclar Vivadent, Lichtenstein) was applied on the restoration margins for cement protection and prevention of the adverse effects of oxygen on cement polymerization.

For cementation of PEEK restorations, the intaglio surface of restorations was first rinsed and dried. Next, the restoration surfaces were sandblasted (Aeroetcher sandblaster, Parkell Inc., NY, USA) with 110 *μ*m aluminum oxide particles with 2.8 bar pressure from 10 mm distance perpendicular to the intaglio surface for 10 s [20]. Tooth preparation and cementation were performed as explained for the LS_2_ restorations.

### 2.4. Cyclic Fatigue Resistance Analysis

To simulate the clinical oral environment, the teeth underwent 15,000 thermal cycles between 5°C and 55°C with a dwell time of 30 s and a transfer time of 10 s [21]. After completion of thermocycling, the teeth underwent 600,000 occlusal load cycles corresponding to 2.5 years of clinical service [22].

A chewing simulator CS-4 (SD Mechatronik GMBH, Feldkirchen-Germany) was used for this purpose which applied 100 N compressive load vertically with 4 Hz frequency. The teeth were immersed in water (37°C) during the study. The teeth were then inspected under a light microscope to detect and exclude the fractured or debonded samples. The samples were then transferred to a universal testing machine (Zwick Roell, Germany) and subjected to a 50 kN load by a metal ball at a crosshead speed of 0.5 mm/minute. The central fossa of the restorations was loaded along the longitudinal axis of the tooth until the restoration failed (fracture of the restoration or tooth or both, or debonding). The load causing endocrown failure was recorded as the PFR of the respective restoration.

### 2.5. Microscopic Analysis

The teeth were inspected under a stereomicroscope (Nikon SMZ 1500, Tokyo, Japan) at x15 magnification. Failure mode was categorized as follows [23]:

• Type 1: debonding of endocrown
• Type 2: fracture of endocrown
• Type 3: fracture in tooth/endocrown complex above the CEJ
• Type 4: fracture in tooth/endocrown complex below the CEJ

In this classification, fracture types 1, 2, and 3 were considered as repairable, and type 4 was regarded as irreparable fracture.

### 2.6. Statistical Analysis

Data were analyzed by SPSS version 23 using descriptive and inferential statistics. The study groups were compared using independent *t*-test and also chi-square and Mann–Whitney tests. The frequency of different modes of failure was compared between the two groups by Fisher's exact test (alpha < 0.05).

## 3. Results

None of the teeth broke after thermocycling and cyclic loading. PFR was recorded as the highest mean load causing failure in Newtons (Table 3). The mean PFR was 6468.89 ± 991.40 N in the PEEK group and 2274.15 ± 510.55 N in the LS_2_ group. The Mann–Whitney test showed a significant difference in PFR of the two groups, and the mean PFR of the PEEK group was significantly higher than that of LS_2_ group (*α* < 0.001).

**Figure 2:**
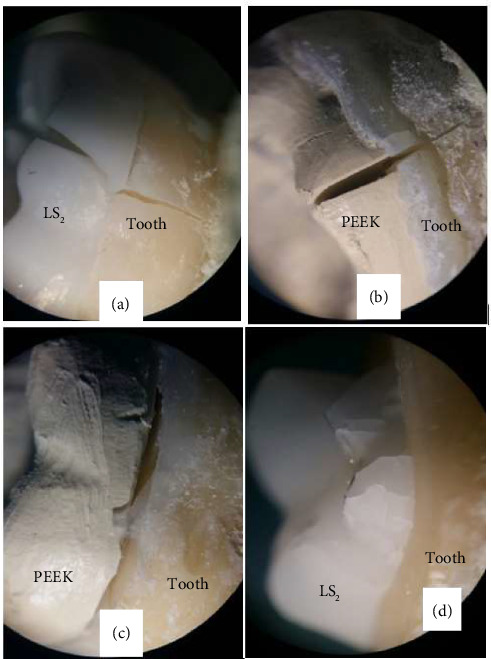
Failure modes. (a) Type 4, LS_2_. (b) Type 4, PEEK. (c) Type 1, PEEK. (d) Type 2, LS_2_.

There were 7 cases of type 4 failure and 3 cases of type 2 failure in the LS_2_ group and 7 cases of type 4 failure and 3 cases of type 1 failure in the PEEK group. The frequency of different failure modes was significantly different between the PEEK and LS_2_ groups (Table 4). In other words, mode of failure had a significant correlation with the study group (*α* < 0.05). Type 4 failure had the highest frequency in both groups (70% of failures in each group).  Figure 2 shows some types of failures in both groups.

## 4. Discussion

The present study assessed the PFR of 20 mandibular first molars restored with LS_2_ and PEEK endocrowns. Evidence shows that endocrowns are a suitable option for reconstruction of endodontically treated posterior teeth, particularly molars [24]. Larger and deeper pulp chamber of molar teeth increases the surface area required for adhesion, which significantly improves the retention of restoration and enables better distribution of masticatory forces, contributing to restoration success [25].

To better simulate the oral clinical environment in the present study, the teeth first underwent thermocycling and were then subjected to cyclic loading. The parameters for these procedures were selected according to the previous studies [26, 27]. According to the findings of Gale and Darvell [28], 10,000 thermocycling is equivalent to one year of in vivo service. Thermocycling can significantly reduce the fracture resistance of zirconia restorations [29]. None of the teeth broke after 15,000 thermal cycles corresponding to 1.5 years of clinical service and 600,000 load cycles corresponding to 2.5 years of clinical function [26, 27]. The reason can be the optimal preparation of teeth, adequate occlusal reduction, and selection of teeth with 4 mm pulp chamber depth. Proper restoration design can effectively decrease the applied stresses and increase the fracture resistance of restorations [30, 31]. The results of PFR testing in a universal testing machine in the present study revealed significantly higher PFR (compressive load at failure point) of PEEK group compared with LS_2_ group, which was in agreement with the results of Ghajghouj and Taşar-Faruk [19], who showed that crowns fabricated from PEEK had the highest fracture resistance compared with those fabricated from other materials such as IPS e.max and zirconia-reinforced LS_2_ ceramics. However, the present results were in contrast to the findings of Elashmawy et al. [32], who showed that the fracture resistance of PEEK was lower than IPS e.max. This difference in the results may be due to the fabrication of endocrowns by the layering technique (1 mm cut back and then veneering) in their study. The PEEK polymer used in this study was BioHPP, which is a modified type of PEEK, containing 20% ceramic filler. Fillers 0.3 to 0.5 *μ*m in size that are homogenously distributed in the polymer matrix can improve the mechanical properties [33]. Also, PEEK has a modulus of elasticity close to that of dentin. Due to this property, it absorbs the stresses and results in their more uniform distribution. In fact, it serves as a stress breaker [34]. The elastic modulus of BioHPP is 4 GPa, which is significantly lower than that of IPS e.max, i.e., 95 GPa, and is closer to that of dentin (13 GPa) [35–37]. The high elastic modulus of IPS e.max is due to the presence of needle-like shaped crystals of LS_2_ in this material [38]. Aside from the elasticity modulus, adhesion also plays a role in fracture resistance [39]. LS_2_ has an optimal bond strength to tooth structure due to the presence of a glass phase in its composition. Bond strength is enhanced by the use of hydrofluoric acid etchant and subsequent application of silane and resin cement [40]; in contrast, no consensus has reached on using a certain material for bonding of PEEK to tooth structure. The hydrophobic nature and low energy level of PEEK compromise its optimal adhesion to dentin [41]. There is some controversy about the best surface treatment. A recent review study reported that application of sulfuric acid and sandblasting along with the application of bonding agents containing methyl methacrylate or pentaerythritol triacrylate was the most efficient method for bonding to PEEK [42]. However, Soares et al. [43] showed that air abrasion is the only method to improve bond adhesion to PEEK. Sandblasting and methacrylate cement were used in the present study for bonding of PEEK endocrowns. Debonding of endocrown or type I failure was only observed in PEEK endocrowns in the present study, which may indicate their lower bond strength. On the other hand, lower modulus of elasticity of PEEK moves the stress concentration away from dentin but shifts it toward the tooth-endocrown interface and results in debonding [44]. Debonding failure was not seen in LS_2_ group in the present study. Although LS_2_ ceramic has the advantage of optimal bond strength to tooth structure by using resin cement, it showed lower PFR than PEEK in the present study, which may indicate the more important role of having a Young's modulus close to that of dentin in increasing the fracture resistance [35, 36]. Type 4 was the most frequent mode of failure in both groups, which is a catastrophic and irreparable failure. Despite the lower elastic modulus of BioHPP polymer and optimal adhesion of LS_2_ that contribute to prevention of fracture through crack bridging, none of the tested materials could prevent irreparable failure. Several papers have shown that the rate of catastrophic failure of PEEK restorations is lower than that of other ceramics due to its lower Young's modulus [35, 45]. However, Aher et al. [46] showed that both PEEK and lithium silicate endocrowns had predominantly catastrophic failures and that there were no significant differences in the frequency of fracture type between the groups.

It appears that restoration design plays an important role in this regard. A previous study showed that extension of restoration to the pulp chamber space affected the mode of failure [30]. Although 4 mm of extension into the pulp chamber can increase the fracture resistance of restoration, it would result in irreparable fracture in case of failure. Nonetheless, this finding does not appear to be clinically important since the loads applied to restorations in the universal testing machine were much greater than those applied to restorations during mastication [30].

A finite element study on the effect of pulp chamber depth on fracture resistance of endocrowns revealed maximum stress accumulation in the axiopulpal line angle upon load application [47]. This type of stress can cause type 4 fracture, i.e., fracture below the CEJ, which was in line with the present findings. Another study demonstrated that the majority of endocrowns fabricated from IPS e.max had fractures below the CEJ upon the application of axial and lateral loads [48], which was in agreement with the present results. The debonding mode of failure in PEEK endocrowns had a low frequency in the study by Ghajghouj and Faruk [19]. Nonetheless, their results cannot be directly compared with the present findings due to using a different classification system. Unlike the present study, all PEEK failures were repairable in the study by Elashmawy et al. [32]. Difference between their results and the present findings may be due to the 3 mm pulp chamber depth in their study versus 4 mm in the present study. It should be noted that although the assessments revealed no cracks in the first phase of the study and prior to PFR analysis in the universal testing machine in the present study, some microcracks might have been missed since they cannot be detected under a light microscope, and their detection requires high-magnification microscopic assessments such as electron microscopy.

### 4.1. Limitations of the Study

It was not feasible to utilize a chewing simulator with a force load exceeding 100 N or periodic forces. It would be more advantageous to conduct this study on alternative types of commercial PEEK polymers currently available in the dental market. The recommended bonding systems, based on the published studies, should be employed for cementation of PEEK.

## 5. Conclusion

Both materials tolerated the fatigue cycles well. The PFR of PEEK endocrowns was significantly higher than that of LS_2_ endocrowns. The majority of failures in both groups were irreparable. In total, the present results indicated that PEEK polymer may be a suitable alternative to LS_2_ for the fabrication of endocrowns.

## Figures and Tables

**Table 1:** Materials used in the study.

Material	Manufacturer	Brand	Composition
Polyether ether ketone	Bredent GmbH & co. KG., Senden, Germany	breCAM.BioHPP	Partially crystalline, thermoplastic, high-performance polymer, 20% ceramic filler (grain size 0.3–0.5 *μ*m)
LS_2_	Ivoclar Vivadent, Schaan, Liechtenstein	IPS e.max CAD	Lithium disilicate–based ceramic: silicon dioxide 57–80 wt%, lithium oxide 11–19 wt%, potassium oxide 0–13 wt%, phosphorus oxide 0–11 wt%, other oxides (ZrO2, ZnO,…)
Dual cure resin cement	Ivoclar Vivadent, Schaan, Liechtenstein	Multilink N	bis-GMA, urethane dimethacrylate and triethylene glycol dimethacrylate, fillers (73.4 wt%): barium glass, ytterbium trifluoride, Ba-Al-fluorosilicate glass, and spheroid mixed oxide (mean particle size: 0.7 *μ*m), initiators, stabilizers, pigments
Primer	Ivoclar Vivadent, Liechtenstein	Multilink N primer A and B	Primer A: aqueous solution of initiators Primer B: HEMA, phosphonic acid, methacrylate monomers
Silane	Ivoclar Vivadent, Liechtenstein	Monobond N	Alcohol solution of silane methacrylate, phosphoric acid methacrylate, and sulphide methacrylate
Porcelain etch	Ivoclar Vivadent, Liechtenstein	IPS ceramic etching gel	5% hydrofluoric acid
Sealer	Dentsply, DeTrey, Konstanz, Germany	AH26	Powder: silver, bismuth oxide, hexamethylenetetramine, titanium oxide Liquid: bisphenol diglycidyl ether
Resin-modified glass ionomer	GC Corporation, Tokyo, Japan	Fuji II LC	Powder: fluoroaluminosilicate glass Liquid: poly acrylic acid, water, hydroxyethyl methacrylate, dimethacrylate, camphorquinone,…

**Table 2:** Firing program for IPS e.max CAD.

Entry time (min)	Entry temp (°C)	Heating rate	Final temp (°C)	Holding time (min)	Start vacuum (°C)	Release vacuum (°C)
6	400	30°C/min	845	10	550	845

**Table 3:** Load at failure point.

Groups	Load at fracture in Newtons (mean ± SD)	
LS_2_	2274 ± 510	*α* < 0.001
PEEK	6468 ± 991	

**Table 4:** The results of failure modes.

Failure mode	LS_2_ (%)	Peak (%)	
Type 1	0	30	*α* < 0.05
Type 2	30	0	
Type 3	0	0	
Type 4	70	70	

## Data Availability

The data supporting the findings of this study are available within the article.
